# Relationship between Uric Acid Level and Severity of Acute Primary Cerebral Infarction: A Cross-Sectional Study

**DOI:** 10.1155/2020/2310307

**Published:** 2020-06-22

**Authors:** Ruying Wang, Yi Zhong, Quan Zhou, Ping Xu

**Affiliations:** ^1^Nanhua University, Hengyang, Hunan, China; ^2^Department of Neurology, The First People's Hospital of Changde City, Changde, Hunan, China; ^3^Ministry of Science and Education, The First People's Hospital of Changde City, Changde, Hunan, China

## Abstract

Numerous studies have shown that uric acid (UA) is associated with cerebrovascular disease, but whether UA is a protective factor or worsens the risk of developing cerebrovascular disease remains controversial. This study investigated the relationship between UA levels detected at admission and the severity of acute primary cerebral infarction. This cross-sectional study enrolled patients with acute primary cerebral infarction (*N* = 238, 157 men). We designated the levels of serum UA measured at the time of admission as the independent variable and the degree of neurological impairment at admission as the dependent variable. The National Institutes of Health Stroke Scale (NIHSS) was used to assess the extent of neurological dysfunction: NIHSS ≤ 5, minor stroke; NIHSS > 5, moderate to severe stroke. There was a statistically significant difference in UA levels between patients with mild cerebral infarctions (NIHSS ≤ 5) and those with moderate or severe cerebral infarctions (NIHSS > 5) (*P* < 0.0001). After adjusting for confounding factors, the serum UA level was found to be nonlinearly related to NIHSS, and the inflection point was 372 *μ*mol/L. The extent of the influence and confidence interval was 0.99 (0.98, 0.99) on the left side of the inflection point and 1.00 (1.00, 1.01) on the right side. There was a nonlinear relationship between the UA level measured on admission and the degree of neurological impairment in patients with acute primary cerebral infarction. When UA was <372 *μ*mol/L, it was negatively correlated with the degree of neurological impairment in patients with acute cerebral infarction, but when UA was ≥372 *μ*mol/L, the protective effect of UA disappeared.

## 1. Introduction

With its highest incidences in Asia—especially in China—and Eastern Europe, cerebral infarction remains a global concern. Moreover, according to the European Stroke Burden Report, no changes in the current incidence of cerebral infarctions will increase the total number of stroke events by more than 30% between 2015 and 2035 [1, 2]. However, the mechanism underlying the onset of cerebral infarction remains to be elucidated. Most studies of cerebral infarction have focused on its treatment and prognosis [3]. Some studies suggest that the neuroendocrine status is related to the prognosis of patients with acute cerebral infarction [4]. Patients with severe neuroendocrine disorders have a poor prognosis. Studies have shown that serum uric acid (UA), a metabolite of purine [5], can predict cardiovascular and cerebrovascular accidents and is associated with early changes in neurological function. A meta-analysis found that high serum UA levels had a neuroprotective effect on the prognosis of patients with acute ischemic stroke [6]. However, the relationship between UA and cerebral infarction is still controversial. While some research has shown that UA is involved in the formation of cerebral atherosclerosis, is a risk factor for cerebral infarction, and can cause cardiovascular and cerebrovascular accidents [7, 8], a study has shown that serum UA has no relationship to cerebral infarction [9]. To help resolve this controversy, the present study investigated whether UA levels at admission were independently associated with the severity of acute primary cerebral infarction.

## 2. Materials and Methods

### 2.1. Research Design

This cross-sectional study explored the relationship between UA and the severity of acute primary cerebral infarction. The level of serum UA at the time of admission was selected as the independent variable and the degree of neurological impairment at admission as the dependent variable.

### 2.2. Research Population

We collected baseline data from patients who were hospitalized in the Department of Neurology, the First People's Hospital of Changde City, from November 1, 2018, to August 31, 2019. The criteria for inclusion in this study were as follows: (1) incidence time < 48 hours and (2) an acute cerebral infarction that was confirmed by MRI during hospitalization. Exclusion criteria were as follows: (1) a history of cerebral infarction, malignant tumor, gouty arthritis, kidney disease, systemic infection, or an autoimmune disease; (2) the recent intake of drugs related to UA metabolism; and (3) missing data. All data were obtained from the participating hospital's electronic medical record system.

### 2.3. Method for Measuring the Degree of Neurological Dysfunction

The National Institutes of Health Stroke Scale (NIHSS) was used to assess the extent of neurological dysfunction. The NIHSS value was assessed and recorded by a neurologist at the time of admission. An NIHSS score of ≤5 was considered to indicate a minor stroke; NIHSS scores of >5, moderate to severe stroke [10–12].

### 2.4. Measurement of UA

The specific process of UA measurement was as follows: (1) Venous blood was collected on the first day of the patient's admission and sent to the laboratory through our hospital's transportation system. (2) UA activity was measured using an oxidase method UA kit, the performance of which was approved by the national testing agency.

### 2.5. Statistical Analysis

We represent continuous variables in two ways. The continuous distribution of normal variables is expressed as the mean ± standard deviation, while skewed distributions are expressed as medians and interquartile ranges (Q1-Q3). Classification variables are expressed in terms of frequency or percentage. We used a one-way analysis of variance (normal distribution), the Kruskal-Wallis H (skewed distribution) test, and the chi-square test (categorical variables) to examine differences between different UA groups. Our method of data analysis can be divided into two steps. In the first step, we explored the linear relationship between UA and NIHSS by using three linear regression models: Model 1, without covariate adjustments; Model 2, adjusted for social demographic data only, including age and sex; and Model 3, adjusted for all the covariates shown in Table 1. In the second step, we explored the nonlinear relationship between UA and NIHSS and performed a smooth curve fit. If nonlinearity was detected, we first calculated the inflection point using a recursive algorithm and then constructed a two-stage linear regression on both sides. The optimal fit model was determined based on the log likelihood ratio test value. To ensure the robustness of the data analysis, we conducted a sensitivity analysis. We converted the UA into a categorical variable and calculated the *P* value of the trend. The purpose of the test was to verify the results of treating UA as a continuous variable and to determine the possibility of nonlinearity. All the analyses were performed with the statistical software package R (http://www.R-project.org, the R Foundation) and EmpowerStats (http://www.empowerstats.com, X&Y Solutions, Inc., Boston, MA). *P* values of <0.05 (both sides) were considered statistically significant.

## 3. Results

### 3.1. Participant Selection

**Figure 1:**
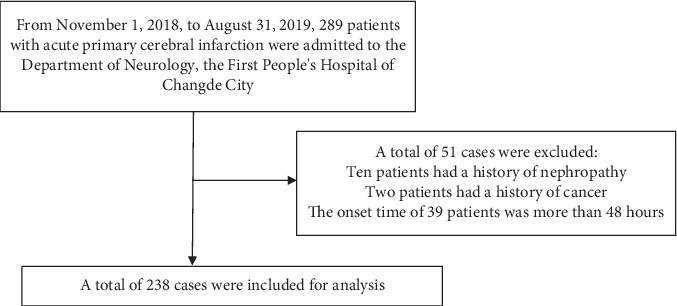
A flow chart.

Based on our strict screening criteria, a total of 238 patients were selected for final data analysis. A total of 51 were excluded, including 10 patients with a history of kidney disease, two patients with a history of cancer, and 39 patients with an onset time of more than 48 hours (see Figure 1 for a flow chart).

### 3.2. Baseline Characteristics of Participants

The baseline data characteristics of the patients are shown in Table 2. The average age of the 238 subjects was 64.41 ± 10.76 years, 65.97% of whom were men. There was statistically significant difference in triglyceride, creatinine, homocysteine, sex, smoking, and NIHSS across quartiles of UA (all *P* < 0.05), but no difference in age, systolic blood pressure, diastolic blood pressure, total cholesterol, LDL cholesterol, HDL cholesterol, urea nitrogen, history of hypertension, history of coronary heart disease, history of diabetes, and drinking (all *P* > 0.05).

### 3.3. Univariate Analysis of the Degree of Neurological Dysfunction

The results of the univariate analysis are listed in Table 3. The univariate analysis showed that sex; systolic blood pressure; diastolic blood pressure; histories of hypertension, diabetes, smoking, and drinking; total cholesterol; triglyceride; high-density lipoprotein; low-density lipoprotein; homocysteine; and creatinine were not associated with NIHSS. Age, urea nitrogen, and history of coronary heart disease were positively correlated with NIHSS, while UA negatively correlated with NIHSS.

### 3.4. The Relationship between UA and NIHSS according to Three Linear Regression Models

The results obtained from the adjusted and unadjusted models are presented in Table 1, which reveals that UA raw data and UA increased by 10 *μ*mol/L. The finding can be explained as follows: the use of the unadjusted model (crude model) indicated an 8% reduction in the risk of moderate to severe cerebral infarction (OR 0.92, 95% CI 0.88 to 0.95, *P* < 0.0001) for every 10 *μ*mol/L increase in UA. In the minimal adjustment model (Model I), the results were interpreted as an 8% reduction in the risk of moderate to severe cerebral infarction (OR 0.92, 95% CI 0.89 to 0.95, *P* < 0.0001) for every 10 *μ*mol/L increase in UA. Finally, after we completely adjusted the variables (Model II), the results could be explained by a 9% increase in the risk of moderate to severe cerebral infarction for each UA increase of 10 *μ*mol/L (OR 0.91, 95% CI 0.87 to 0.95, *P* < 0.0001). For the sensitivity analysis, we also treated UA as a quarantine (quartile) and observed the same trend. As UA concentration increased, the risk of moderate to severe cerebral infarction decreased.

### 3.5. The Analyses of Nonlinear Relationships

**Figure 2:**
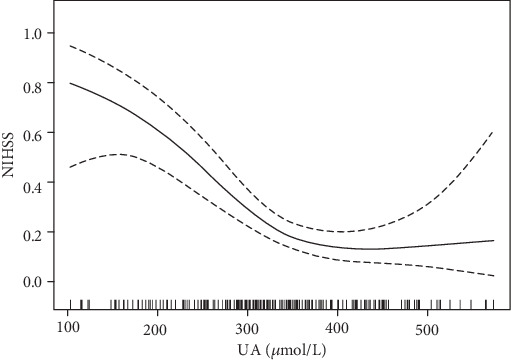
Relationship between UA (*μ*mol/L) and NIHSS. The middle line represents a smooth curve fit between the variables. The curves on both sides represent the 95% confidence interval for the fit. The model was adjusted according to sex, age, systolic BP, diastolic BP, hypertension, diabetes, coronary artery disease, smoking, drinking, total cholesterol, triglyceride, HDL cholesterol, LDL cholesterol, homocysteine, creatinine, and urea nitrogen.

This study found a nonlinear relationship between UA and NIHSS (adjusted sex, age, systolic blood pressure, diastolic blood pressure, hypertension, diabetes, coronary artery disease, smoking, alcohol consumption, total cholesterol, triglycerides, high-density lipoprotein cholesterol, LDL cholesterol, homocysteine, creatinine, and urea nitrogen; Figure 2). Through the two-segment linear regression model, the inflection point was calculated to be 372 *μ*mol/L, and the influence and confidence intervals of the left and right sides of the inflection point were determined to be 0.99 (0.98, 0.99) and 1.00 (1.00, 1.01) (Table 4), respectively.

## 4. Discussion

This study suggested that when UA was <372 *μ*mol/L, UA was negatively correlated with the degree of neurological damage in patients with acute cerebral infarction. Yang et al. [13] found UA concentration to be negatively correlated with NIHSS scores. Two animal experiments found that treatment with UA after cerebral ischemia could maintain a high perfusion state and reduce nerve damage [14, 15]. Chiquete et al. [16] posited that the correlation between low concentration of UA and severity of acute cerebral infarction could be used as a marker of the degree of cerebral infarction. According to Arevalo-Lorido et al. [17], NIHSS scores decrease with increasing UA, even in patients with cerebral infarction and abnormal renal function. In performing a randomized, double-blind, placebo-controlled phase 2b/3 trial in which UA was added during thrombolytic therapy administered to patients with acute cerebral infarction, Chamorro et al. [18] found that UA had a protective effect on the patient's nerve function. However, other studies have yielded different results. Sarfo et al. [19] found that UA concentration was positively correlated with the severity of cerebral infarction and positively correlated with early mortality in patients with acute cerebral infarction. Chen et al. [20] found that serum UA level was not significantly correlated with the prognosis of patients with cerebral infarction. The wide variability in these reports can be attributed to different selected outcome indicators, research populations, and research types. Hence, comparisons among these findings are difficult.

The neuroprotective effects of UA can be explained in the following ways: (1) UA has an antioxidative effect, preventing the cerebrovascular system from being damaged by oxidative stress [21, 22]. (2) UA can remove oxidative free radicals caused by chelation of metal ions and increase the metabolism of purine in the brain [23]. (3) UA protects local cerebral blood supply by activating the expression of neurotrophic factors and reduces brain damage caused by reperfusion [24, 25]. It has been suggested in the literature that UA is an endogenous antioxidant and that low levels of UA are associated with the increased prevalence of neurodegenerative and inflammatory diseases of the central nervous system and deterioration of clinical processes. UA is rapidly consumed after acute cerebral infarction, and high levels of UA are associated with better prognoses [26]. This observation is consistent with our research conclusions.

Many previous studies have revealed a relationship between UA and the severity of cerebral infarction, and our study provides supporting evidence for the protective effect of UA on nerve function in patients with acute cerebral infarction. In this study, the nonlinear relationship between UA and the severity of cerebral infarction was determined, curve fitting was carried out, and the inflection point was calculated. The results were intuitive. It was found that a correlation remains, despite the adjustment of many confounding factors. A meta-analysis of 12739 patients with acute cerebral infarction suggested that, for patients with acute cerebral infarction, high levels of UA at onset have neuroprotective effects and are markers of a better prognosis, especially for patients undergoing thrombolytic therapy, regardless of the type of thrombolytic therapy [6]. This is basically consistent with the results of our study. It is necessary to consider whether patients with cerebral infarction treated with thrombolytic therapy could be given UA supplement, maintaining the upper limit of serum UA < 372 *μ*mol/L, to minimize neurological damage. In particular, UA supplementation may have greater clinical significance in patients with acute episodes who cannot be treated with thrombolytic therapy. This will be our next research direction.

This study also has several limitations: (1) It was small, mainly comprising patients with acute primary cerebral infarction, and all participants were Chinese. (2) As a cross-sectional study, it could not provide evidence of causality. Large prospective cohort studies are needed to investigate this. (3) Previous studies have shown that UA may be related to the size, type, and location of cerebral infarction [5, 27]. Studies have suggested that UA levels may be associated with secondary epilepsy following cerebral infarction [28], but these data were not collected in this study. (4) Patients with cerebral infarction may also have Parkinson's disease, decreased cognitive function, or other chronic diseases, which may also be related to UA levels. Due to the limitations of the original data, this avenue of research could not be further explored [29–31].

## 5. Conclusion

There was a nonlinear relationship between the UA level measured on admission and the degree of neurological impairment in patients with acute primary cerebral infarction. When UA was <372 *μ*mol/L, it was negatively correlated with the degree of neurological impairment in patients with acute cerebral infarction, but when UA was ≥372 *μ*mol/L, the protective effect of UA disappeared.

## Figures and Tables

**Table 1:** Relationship between UA and NIHSS in different models.

Variable	Crude model		Model I		Model II	
	OR (95% CI)	*P* value	OR (95% CI)	*P* value	OR (95% CI)	*P* value
UA (*μ*mol/L)	0.99 (0.99, 0.99)	<0.0001	0.99 (0.99, 1.00)	<0.0001	0.99 (0.99, 0.99)	<0.0001
UA (10 *μ*mol/L)	0.92 (0.88, 0.95)	<0.0001	0.92 (0.89, 0.95)	<0.0001	0.91 (0.87, 0.95)	<0.0001
UA (quartile)						
Q1	Reference		Reference		Reference	
Q2	0.44 (0.21, 0.94)	0.0337	0.46 (0.21, 0.98)	0.0452	0.44 (0.18, 1.04)	0.0602
Q3	0.13 (0.05, 0.34)	<0.0001	0.14 (0.05, 0.35)	<0.0001	0.13 (0.04, 0.37)	0.0002
Q4	0.15 (0.06, 0.37)	<0.0001	0.17 (0.07, 0.42)	0.0001	0.14 (0.05, 0.42)	0.0004
*P* for trend		<0.0001		<0.0001		<0.0001

Abbreviations: CI: confidence interval. Model I adjusted for sex and age; Model II adjusted for sex, age, systolic BP, diastolic BP, hypertension, diabetes, coronary artery disease, smoking, drinking, total cholesterol, triglyceride, HDL cholesterol, LDL cholesterol, homocysteine, creatinine, and urea nitrogen.

**Table 2:** Baseline characteristics of the participants (*N* = 238).

Characteristic	UA quartiles (*μ*mol/L)				*P* value
	Q1 (103-256)	Q2 (262-322)	Q3 (323-384)	Q4 (387-573)	
No. of participants	58	59	57	64	
Age (years, median, Q1-Q3)	67.50 (56.25-73.75)	64.00 (55.00-74.00)	65.00 (57.00-71.00)	62.00 (53.00-69.00)	0.182
Systolic BP (mmHg, mean ± sd)	160.71 ± 25.80	155.22 ± 22.96	155.47 ± 20.47	161.81 ± 23.60	0.271
Diastolic BP (mmHg, median, Q1-Q3)	86.00 (78.25-98.75)	85.00 (78.00-95.50)	84.00 (78.00-94.00)	88.00 (79.00-100.25)	0.266
Total cholesterol (mmol/L, median, Q1-Q3)	4.50 (3.87-5.00)	4.64 (4.21-5.37)	4.48 (4.20-4.93)	4.80 (4.08-5.40)	0.521
Triglyceride (mmol/L, median, Q1-Q3)	1.39 (0.98-1.99)	1.44 (1.11-1.97)	1.80 (1.21-2.45)	1.67 (1.36-2.76)	0.009
HDL cholesterol (mmol/L, median, Q1-Q3)	1.12 (0.93-1.31)	1.09 (0.94-1.28)	1.02 (0.91-1.15)	1.03 (0.95-1.17)	0.107
LDL cholesterol (mmol/L, median, Q1-Q3)	2.51 (2.09-3.20)	2.60 (2.25-3.40)	2.64 (2.39-2.98)	2.70 (2.25-3.21)	0.772
Creatinine (*μ*mol/L, median, Q1-Q3)	66.00 (54.00-75.00)	69.00 (60.00-85.00)	76.00 (67.00-88.00)	87.50 (70.00-104.00)	<0.001
Urea nitrogen (*μ*mol/L, median, Q1-Q3)	5.42 (4.56-6.46)	5.45 (4.59-6.32)	5.51 (4.41-6.45)	5.33 (4.39-6.37)	0.944
Homocysteine (*μ*mol/L, median, Q1-Q3)	11.64 (8.90-14.28)	12.00 (9.87-14.61)	12.91 (10.40-15.30)	13.05 (10.87-16.91)	0.015
Sex (*n*, %)					<0.001
Male	27 (46.55%)	38 (64.41%)	39 (68.42%)	53 (82.81%)	
Female	31 (53.45%)	21 (35.59%)	18 (31.58%)	11 (17.19%)	
Hypertension (*n*, %)					0.337
No	25 (43.10%)	24 (40.68%)	16 (28.07%)	26 (40.62%)	
Yes	33 (56.90%)	35 (59.32%)	41 (71.93%)	38 (59.38%)	
Diabetes (*n*, %)					0.994
No	49 (84.48%)	50 (84.75%)	48 (84.21%)	55 (85.94%)	
Yes	9 (15.52%)	9 (15.25%)	9 (15.79%)	9 (14.06%)	
Coronary artery disease (*n*, %)					0.096
No	46 (79.31%)	54 (91.53%)	53 (92.98%)	57 (89.06%)	
Yes	12 (20.69%)	5 (8.47%)	4 (7.02%)	7 (10.94%)	
Smoking (*n*, %)					0.013
No	45 (77.59%)	44 (74.58%)	35 (61.40%)	34 (53.12%)	
Yes	13 (22.41%)	15 (25.42%)	22 (38.60%)	30 (46.88%)	
Drinking (*n*, %)					0.111
No	53 (91.38%)	44 (74.58%)	46 (80.70%)	50 (78.12%)	
Yes	5 (8.62%)	15 (25.42%)	11 (19.30%)	14 (21.88%)	
NIHSS					<0.001
Minor stroke (NIHSS ≤ 5)	28 (48.28%)	40 (67.80%)	50 (87.72%)	55 (85.94%)	
Moderate to severe stroke (NIHSS > 5)	30 (51.72%)	19 (32.20%)	7 (12.28%)	9 (14.06%)	

Note: the continuous variable of nonnormal distribution is calculated by Kruskal-Wallis H test. BP: blood pressure; HDL: high-density lipoprotein; LDL: low-density lipoprotein; UA: uric acid; NIHSS: National Institutes of Health Stroke Scale.

**Table 3:** Univariate analysis for NIHSS.

Covariate	Statistics	OR (95% CI)	*P* value
Sex (*n*, %)			
Male	157 (65.97%)	Ref	
Female	81 (34.03%)	1.71 (0.95, 3.08)	0.0726
Age (years)	64.41 ± 10.75	1.04 (1.01, 1.07)	0.0064
Systolic BP (mmHg)	158.39 ± 23.35	0.99 (0.98, 1.00)	0.2146
Diastolic BP (mmHg)	87.84 ± 15.03	0.99 (0.97, 1.01)	0.2367
Hypertension (*n*, %)			
No	91 (38.24%)	Ref	
Yes	147 (61.76%)	0.76 (0.42, 1.35)	0.3468
Diabetes (*n*, %)			
No	202 (84.87%)	Ref	
Yes	36 (15.13%)	1.88 (0.90, 3.95)	0.0940
Coronary artery disease (*n*, %)			
No	210 (88.24%)	Ref	
Yes	28 (11.76%)	5.22 (2.29, 11.89)	<0.0001
Smoking (*n*, %)			
No	158 (66.39%)	Ref	
Yes	80 (33.61%)	0.62 (0.33, 1.17)	0.1373
Drinking (*n*, %)			
No	193 (81.09%)	Ref	
Yes	45 (18.91%)	0.83 (0.39, 1.76)	0.6321
Total cholesterol (mmol/L)	4.70 ± 1.02	0.89 (0.67, 1.18)	0.4218
Triglyceride (mmol/L)	1.94 ± 1.25	0.72 (0.53, 0.96)	0.0528
HDL cholesterol (mmol/L)	1.10 ± 0.25	2.09 (0.68, 6.48)	0.2002
LDL cholesterol (mmol/L)	2.74 ± 0.80	0.91 (0.63, 1.32)	0.6152
Homocysteine (*μ*mol/L)	13.90 ± 7.82	0.97 (0.92, 1.02)	0.2616
Creatinine (*μ*mol/L)	78.90 ± 28.88	1.01 (1.00, 1.01)	0.2593
Urea nitrogen (*μ*mol/L)	5.64 ± 1.94	1.23 (1.06, 1.43)	0.0079
UA (*μ*mol/L)	328.87 ± 98.78	0.99 (0.99, 0.99)	<0.0001

Abbreviations: CI: confidence interval; OR: odds ratio.

**Table 4:** Univariate analysis for NHISS.

Inflection point of UA	Effect size (OR)	95% CI *P* value	*P* value
<372	0.99	0.98 to 0.99	<0.0001
≥372	1.00	1.00 to 1.01	0.3487

Abbreviations: CI: confidence interval; OR: odds ratio.

## Data Availability

The data sets used and/or analyzed in this study may be obtained by appropriate authorization or reasonable request.
